# Targeting adhesion to the vascular niche to improve therapy for acute myeloid leukemia

**DOI:** 10.1038/s41467-020-17594-7

**Published:** 2020-07-23

**Authors:** Myriam L. R. Haltalli, Cristina Lo Celso

**Affiliations:** 1 Department of Life Sciences, Imperial College London, Sir Alexander Fleming Building, SW7 2AZ London, UK; 2 The Francis Crick Institute, WC2A 3LY London, UK; 3 Present Address: Wellcome - Medical Research Council Cambridge Stem Cell Institute, University of Cambridge, Jeffrey Cheah Biomedical Centre, Cambridge Biomedical Campus, CB2 0AW Cambridge, UK

**Keywords:** Cancer microenvironment, Cancer microenvironment, Cancer therapeutic resistance, Cancer therapeutic resistance

## Abstract

Niche hijack by malignant cells is considered to be a prominent cause of disease relapse. Barbier and colleagues uncover (E)-selectin as a novel mediator of malignant cell survival and regeneration which, upon blockade, has the potential to significantly improve therapeutic outcomes.

## Not-so-welcome to the neighborhood

Hematopoietic stem cells (HSCs) sit at the top of the hematological hierarchy as multipotent cells with the capacity to self-renew and give rise to various progenitor populations, which maintain the production of short-lived mature lineages (including blood cells) throughout life. They reside within the bone marrow (BM), within specialized niches that facilitate their survival and regulate their function via local interactions with diverse stromal and hematopoietic cells. It is widely accepted that spatially distinct niches exist to support HSCs with a range of differentiation biases and quiescence states. These are broadly split into the central BM niche, located in the deeper BM and rich in sinusoid capillaries, and the endosteal niche, in close proximity to the bone inner surface and rich in arterioles and transitional vessels that feed into the sinusoidal network^1^. In both locations, HSCs reside in perivascular niches where endothelial cells (ECs) and their associated mesenchymal stem cells critically maintain and regulate them^2^.

Hematological malignancies, such as acute myeloid leukemia (AML), tend to arise from the expansion of transformed hematopoietic stem and progenitor cells (HSPCs) with subclonal genotypes, resulting in refractory clones that are able to resist chemotherapy and ultimately lead to the relapse of disease. Leukemia stem cells (LSCs) share many features with healthy HSCs, which has led to the hypothesis that LSCs similarly reside in, and depend on, specific BM “neighborhoods” that support their expansion and survival^3,4^. There is a growing need for novel therapies to reduce malignancy-associated morbidity and mortality and, due to the fact that many mechanisms of immune evasion and chemoresistance are partly dependant on it, the BM niche represents a unique target to improve treatment outcomes^5^. The recent work by Barbier and colleagues^6^ is therefore timely as it reveals, using AML as a model, a new cell-extrinsic niche-based pathway that directly mediates therapy resistance. The authors propose a method with the potential to improve AML treatment efficacy by targeting the vascular niche.

## Holding on tight

Endothelial (E)-selectins are cell adhesion molecules expressed in the vascular niche, which have well-characterized roles in leukocyte homing. E-selectin is constitutively expressed on BM endothelium, aiding the homing and engraftment of circulating HSPCs that possess the necessary counter-receptors. Previous work had demonstrated that adhesion to E-selectin promotes the proliferation of HSCs, directly triggers their activation and induces lineage commitment^7^. Interestingly, based on earlier observations of E-selectin expression in BM areas where leukemia cells home^8^, the hypothesis arose that LSCs may be protected from the effects of chemotherapy through their anchorage in niches enriched in its expression. The work from Winkler et al. uncovered an unexpected additional role for this adhesion molecule as a therapeutic target when its inhibition induced HSC chemoresistance and radioresistance. Overall, their findings raised the question whether specific binding of tumor cells with endothelium expressing E-selectin might regulate tumor growth and proliferation^7^. Barbier et al. address this question and show that, in the context of disease, interaction with E-selectin plays a direct role in promoting malignant cells’ survival in the BM, a textbook example of niche hijack.

Inflammation is a hallmark of cancer and we have previously hypothesized that it is a key player in driving remodeling of the BM niche during leukemia^9^. E-selectin is expressed by ECs at the leading edge of solid tumors and at pre-metastatic sites, which are also highly inflammatory environments^10,11^. Barbier et al. demonstrate that the inflammation generated by AML in the BM directly drives the increased levels of E-selectin on BM ECs of leukemic mice. This resulted in the postulation that the specific upregulation on tumor-associated vasculature may be less involved in the homing of AML cells, but more so as a factor directly contributing to their survival and regeneration by creating a protective niche for LSCs to hold on to, thus promoting therapy resistance.

## A novel target for improved therapy

While there is an ever-increasing number of targeted therapies to treat leukemia, patient outcome remains poor, thus there is an urgent need to develop new strategies. Over the past few years, with the advance of increasingly sophisticated technologies to study the BM microenvironment, we have gained fascinating insight into this tissue. We can now appreciate that it is a complex and dynamic entity that actively interacts with healthy hematopoietic cells, as well as their malignant counterparts, playing an important role in directing their fates. Our knowledge of how leukemia cells co-opt and alter BM niches offers many opportunities for therapeutic exploitation, and more targeted interventions of novel pathways to restore healthy hematopoiesis and limit disease relapse.

By elegantly demonstrating that the leukemic blasts most likely to survive chemotherapy were those characterized by higher E-selectin binding potential, Barbier and colleagues suggested that these cells were the main contributors to disease relapse. To test the role of E-selectin in supporting malignant cells, they sought to understand the outcome if this adhesion molecule was absent in the BM (using an E-selectin gene knock-out mouse model) or blocked therapeutically. E-selectin can be inhibited by administering a selective small molecule antagonist called GMI-1271 (also known as uproleselan). It has been shown that this treatment effectively displaces chronic myeloid leukemia (CML) cells from the BM endothelium, promoting cell cycle progression and downregulating the expression of the E-selectin ligand CD44—thus reinforcing the reduced adhesion of CML cells to the BM endothelium^12^. Using an innovative method, the authors investigated whether functional LSCs were amongst those protected by E-selectin-mediated interactions in the vascular niche. They developed a quantitative in vivo LSC chemosensitivity assay, based on the principle of limiting dilution transplants widely used for enumerating HSCs. By comparing the number of persisting LSCs in recipients of BM cells from chemotherapy-treated, E-selectin gene-deleted, or GMI-1271-treated wild-type mice, they demonstrated that the deletion or blockade of E-selectin drastically reduced LSC survival. Therefore, E-selectin mediates significant intracellular pro-survival signaling for LSCs, and this unique characteristic was not replicated by other adhesion molecules and chemokines involved in BM retention.

Up until now, combinations of chemotherapy with approaches that target mainly AML-intrinsic mechanisms have been proposed^13^, including the use of CXCR4 antagonists^14^ and anti-inflammatory therapies^15^. With this work, the authors bring to light the fact that cell-extrinsic targets could prove hugely valuable in future phases of therapy development. By administering GMI-1271 alongside a standard chemotherapy regime in leukemic mice, Barbier et al. were able to double the duration of mouse survival over chemotherapy alone. Endogenous, healthy, HSPC populations exhibited strikingly different responses when compared to AML blasts, suggesting that this treatment strategy protects those populations in the BM. These intriguing data demonstrate that therapeutic blocking of this vascular niche-mediated survival pathway could complement conventional AML treatment regimens to improve efficacy and extend overall survival.

## Future directions and clinical impact

We must harness the progress made so far in creating a detailed map of the BM microenvironment with all of its structures and components at steady state, in order to keep elucidating the underlying mechanisms of disease-mediated niche hijack. The number of clinical trials arising from a deeper understanding of the HSC niche is bound to increase. Based on the work presented by Barbier and colleagues, a phase I/II clinical trial is already in progress to evaluate the use of GMI-1271 alongside standard therapy in relapsed/refractory adult AML (NCT02306291). So far, they have shown greater rates of clinical remission and extended median patient survival, which is consistent with the findings in vivo presented by the authors. The future of these “nichotherapies”^16^ to facilitate chemotherapy looks extremely promising given the rise in demand for new agents with good tolerance in a broad range of AML patients, increased efficacy and, most importantly, the ability to limit disease relapse. The work presented here provides hope that innovative methods of targeting malignant BM niches could be the key to making AML a treatable disease.
